# 4-(3-Chloro-2,2-dimethyl­propanamido)­benzene­sulfonamide

**DOI:** 10.1107/S1600536812046806

**Published:** 2012-11-24

**Authors:** Şerife Pınar Yalçın, Mehmet Akkurt, Mustafa Durgun, Baki Türkkan, Hasan Türkmen

**Affiliations:** a Department of Physics, Faculty of Arts and Sciences, Harran University, 63300 Şanlıurfa, Turkey; b Central Research Lab, Harran University, Osmanbey Campus, 63300 Şanlıurfa, Turkey; c Department of Physics, Faculty of Sciences, Erciyes University, 38039 Kayseri, Turkey; d Department of Chemistry, Faculty of Arts and Sciences, Harran University, 63300 Şanlıurfa, Turkey

## Abstract

In the title compound, C_11_H_15_ClN_2_O_3_S, the 3-chloro-2,2-dimethyl­propanamide and sulfonamide substituents are arranged on opposite sides of the benzene ring plane. In the crystal, mol­ecules are linked by N—H⋯O and C—H⋯O hydrogen bonds, forming a three-dimensional network.

## Related literature

For the anti­bacterial, anti­microbial and anti­glaucoma activity of sulfonamides and their derivatives and for their physical properties and pharmacological applications, see: Poulsen *et al.* (2005 ▶); Supuran & Scozzafava (2000 ▶). For related structures, see: Akkurt *et al.* (2010 ▶); Idemudia *et al.* (2012 ▶); Asiri *et al.* (2012 ▶). For the synthesis, see: Türkmen *et al.* (2011 ▶).
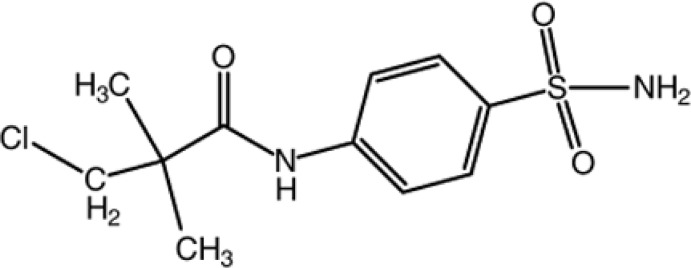


## Experimental

### 

#### Crystal data

C_11_H_15_ClN_2_O_3_S
*M* *_r_* = 290.77
Monoclinic, 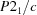

*a* = 20.4359 (11) Å
*b* = 7.2437 (4) Å
*c* = 9.4693 (5) Å
β = 98.222 (3)°
*V* = 1387.35 (13) Å^3^
*Z* = 4
Mo *K*α radiation
μ = 0.43 mm^−1^
*T* = 294 K
0.31 × 0.14 × 0.13 mm

#### Data collection

Rigaku R-AXIS RAPID-S diffractometer
Absorption correction: refined from Δ*F* (*XABS2*; Parkin *et al.*, 1995 ▶) *T*_min_ = 0.931, *T*_max_ = 0.946
4240 measured reflections
4240 independent reflections
2054 reflections with *I* > 2σ(*I*)

#### Refinement

*R*[*F* ^2^ > 2σ(*F* ^2^)] = 0.084
*wR*(*F* ^2^) = 0.231
*S* = 1.02
4240 reflections
175 parameters
2 restraints
H atoms treated by a mixture of independent and constrained refinement
Δρ_max_ = 0.36 e Å^−3^
Δρ_min_ = −0.33 e Å^−3^

### 

Data collection: *CrystalClear* (Rigaku/MSC, 2005 ▶); cell refinement: *CrystalClear*; data reduction: *CrystalClear*; program(s) used to solve structure: *SIR97* (Altomare *et al.*, 1999 ▶); program(s) used to refine structure: *SHELXL97* (Sheldrick, 2008 ▶); molecular graphics: *ORTEP-3 for Windows* (Farrugia, 2012 ▶); software used to prepare material for publication: *WinGX* (Farrugia, 2012 ▶) and *PLATON* (Spek, 2009 ▶).

## Supplementary Material

Crystal structure: contains datablock(s) global, I. DOI: 10.1107/S1600536812046806/sj5282sup1.cif

Structure factors: contains datablock(s) I. DOI: 10.1107/S1600536812046806/sj5282Isup2.hkl

Supplementary material file. DOI: 10.1107/S1600536812046806/sj5282Isup3.cml

Additional supplementary materials:  crystallographic information; 3D view; checkCIF report

## Figures and Tables

**Table 1:** Hydrogen-bond geometry (Å, °)

*D*—H⋯*A*	*D*—H	H⋯*A*	*D*⋯*A*	*D*—H⋯*A*
N1—H1*N*⋯O2^i^	0.88 (3)	2.57 (5)	3.035 (4)	114 (4)
N1—H1*N*⋯O1^ii^	0.88 (3)	2.21 (4)	3.043 (5)	160 (5)
N1—H2*N*⋯O1^iii^	0.88 (2)	2.10 (4)	2.921 (4)	155 (5)
N2—H3*N*⋯O3^iv^	0.91 (6)	2.16 (6)	3.063 (5)	173 (5)
C11—H11*B*⋯O3^iv^	0.97	2.44	3.391 (5)	166

Symmetry codes: (i) 
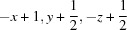
; (ii) 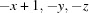; (iii) 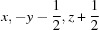; (iv) 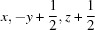.

**Fig. 1.**
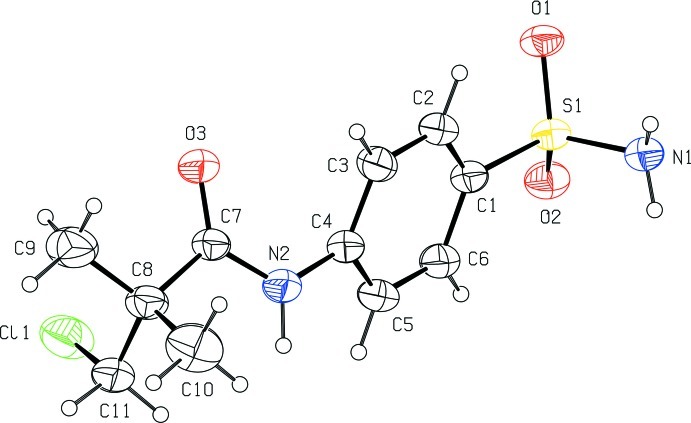
The title molecule with the atom numbering scheme. Displacement ellipsoids for non-H atoms are drawn at the 30% probability level.

**Fig. 2.**
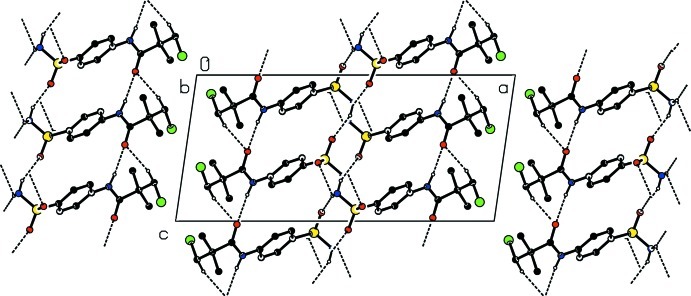
Packing of the title compound viewed along the b axis with N—H···O and C—H···O hydrogen bonds drawn as dashed lines. H atoms not involved in hydrogen bonding are omitted for clarity.

## supplementary crystallographic information

### Comment

Sulfonamides are of interest because of their unique biological properties. They are known inhibitors of the carbonic anhydrase enzyme, currently used for the treatment of glaucoma in clinical medicine (Poulsen *et al.*, 2005; Supuran & Scozzafava, 2000). The design and development of new sulfanilamide derivatives can help determine any structural requirements for improved biological activity. In this study, we have prepared and determined the crystal structure of 4-(3-Chloro-2,2-dimethylpropanoylamino)-benzenesulfonamide (I).

In Fig. 1, the molecular structure of the title compound is not planar. In the 3-chloro-2,2-dimethylpropanamide moiety of (I), the N2—C7—C8—C9, N2—C7—C8—C10 and N2—C7—C8—C11 torsion angles are 178.6 (4), 57.2 (5) and -59.4 (5) °, respectively. The values of the bond lengths and bond angles in (I) are within the normal range and are comparable to those previously reported for the related structures (Akkurt *et al.*, 2010; Idemudia *et al.*, 2012; Asiri *et al.*, 2012). The crystal structure is stabilized by intermolecular N—H···O and C—H···O hydrogen bonds (Table 1 and Fig. 2).

### Experimental

Nucleophilic acyl substitution of 3-chloro-2,2-dimethyl-propanoylchloride with sulfanilamide gave the title compound as described previously (Türkmen *et al.*, 2011). Crystals suitable for X-ray diffraction studies were grown by slow evaporation of an ethanol, chloroform, dichloromethane (4/3/3 *v*/*v*) solution of the product.

### Refinement

The H atoms on the NH and NH_2_ groups were located from a difference Fourier map and refined with distance restraints of N—H = 0.88 (1) Å for the NH_2_, with *U*_iso_(H) = 1.2*U*_eq_(N). The remaining H atoms were positioned geometrically, with C—H = 0.93–0.97 Å, and refined as riding with *U*_iso_(H) = 1.2 or 1.5 *U*_eq_(C). The high R factor, low ratio of observed to unique reflections and relatively high su values indicate that the crystals were of rather poor quality and did not diffract strongly.

### Crystal data

**Table:**

C_11_H_15_ClN_2_O_3_S	*F*(000) = 608
*M**_r_* = 290.77	*D*_x_ = 1.392 Mg m^−^^3^
Monoclinic, *P*2_1_/*c*	Mo *K*α radiation, λ = 0.71073 Å
Hall symbol: -P 2ybc	Cell parameters from 5710 reflections
*a* = 20.4359 (11) Å	θ = 2.5–30.5°
*b* = 7.2437 (4) Å	µ = 0.43 mm^−^^1^
*c* = 9.4693 (5) Å	*T* = 294 K
β = 98.222 (3)°	Needle, white
*V* = 1387.35 (13) Å^3^	0.31 × 0.14 × 0.13 mm
*Z* = 4	

### Data collection

**Table:**

Rigaku R-AXIS RAPID-S diffractometer	4240 independent reflections
Radiation source: Sealed Tube	2054 reflections with *I* > 2σ(*I*)
Graphite Monochromator monochromator	*R*_int_ = 0.000
Detector resolution: 10.0000 pixels mm^-1^	θ_max_ = 30.6°, θ_min_ = 3.0°
ω scans	*h* = −29→28
Absorption correction: part of the refinement model (Δ*F*) (*XABS2*; Parkin *et al.*, 1995)	*k* = 0→10
*T*_min_ = 0.931, *T*_max_ = 0.946	*l* = 0→13
4240 measured reflections	

### Refinement

**Table:**

Refinement on *F*^2^	Primary atom site location: structure-invariant direct methods
Least-squares matrix: full	Secondary atom site location: difference Fourier map
*R*[*F*^2^ > 2σ(*F*^2^)] = 0.084	Hydrogen site location: inferred from neighbouring sites
*wR*(*F*^2^) = 0.231	H atoms treated by a mixture of independent and constrained refinement
*S* = 1.02	*w* = 1/[σ^2^(*F*_o_^2^) + (0.0751*P*)^2^ + 1.0033*P*] where *P* = (*F*_o_^2^ + 2*F*_c_^2^)/3
4240 reflections	(Δ/σ)_max_ < 0.001
175 parameters	Δρ_max_ = 0.36 e Å^−^^3^
2 restraints	Δρ_min_ = −0.33 e Å^−^^3^

### Special details

**Table:**

Experimental. Absorption correction: (XABS2; Parkin *et al.*, 1995) Cubic fit to sin(theta)/lambda - 24 parameters
Geometry. Bond distances, angles *etc*. have been calculated using the rounded fractional coordinates. All su's are estimated from the variances of the (full) variance-covariance matrix. The cell e.s.d.'s are taken into account in the estimation of distances, angles and torsion angles
Refinement. Refinement on *F*^2^ for ALL reflections except those flagged by the user for potential systematic errors. Weighted *R*-factors *wR* and all goodnesses of fit *S* are based on *F*^2^, conventional *R*-factors *R* are based on *F*, with *F* set to zero for negative *F*^2^. The observed criterion of *F*^2^ > σ(*F*^2^) is used only for calculating -*R*-factor-obs *etc*. and is not relevant to the choice of reflections for refinement. *R*-factors based on *F*^2^ are statistically about twice as large as those based on *F*, and *R*-factors based on ALL data will be even larger.

### Fractional atomic coordinates and isotropic or equivalent isotropic displacement parameters (Å^2^)

**Table:**

	*x*	*y*	*z*	*U*_iso_*/*U*_eq_	
Cl1	0.05415 (8)	0.1450 (2)	0.13028 (15)	0.1040 (6)	
S1	0.43958 (5)	−0.22940 (14)	0.07872 (10)	0.0540 (3)	
O1	0.45415 (14)	−0.1736 (4)	−0.0593 (3)	0.0634 (10)	
O2	0.42131 (15)	−0.4163 (4)	0.1004 (3)	0.0684 (11)	
O3	0.19397 (15)	0.3907 (4)	0.0227 (3)	0.0689 (10)	
N1	0.50493 (18)	−0.1856 (5)	0.1885 (3)	0.0592 (11)	
N2	0.22555 (18)	0.2516 (5)	0.2354 (4)	0.0640 (13)	
C1	0.37522 (19)	−0.0863 (6)	0.1199 (4)	0.0550 (14)	
C2	0.3682 (2)	0.0917 (6)	0.0669 (4)	0.0603 (14)	
C3	0.3193 (2)	0.2057 (6)	0.1041 (4)	0.0628 (17)	
C4	0.2770 (2)	0.1406 (6)	0.1935 (4)	0.0579 (14)	
C5	0.2852 (2)	−0.0344 (7)	0.2500 (4)	0.0691 (17)	
C6	0.3339 (2)	−0.1493 (7)	0.2132 (4)	0.0675 (16)	
C7	0.1859 (2)	0.3629 (6)	0.1465 (4)	0.0563 (14)	
C8	0.1294 (2)	0.4544 (6)	0.2119 (4)	0.0643 (16)	
C9	0.0901 (3)	0.5785 (8)	0.1019 (6)	0.102 (3)	
C10	0.1581 (3)	0.5664 (8)	0.3447 (6)	0.102 (3)	
C11	0.0849 (2)	0.3082 (7)	0.2637 (5)	0.0727 (18)	
H1N	0.519 (3)	−0.074 (3)	0.174 (6)	0.1230*	
H2	0.39660	0.13480	0.00580	0.0720*	
H2N	0.500 (3)	−0.209 (8)	0.277 (2)	0.1230*	
H3	0.31500	0.32570	0.06900	0.0750*	
H3N	0.213 (3)	0.216 (8)	0.320 (6)	0.1230*	
H5	0.25770	−0.07550	0.31350	0.0830*	
H6	0.33890	−0.26790	0.25080	0.0810*	
H9A	0.11770	0.67740	0.07780	0.1520*	
H9B	0.05290	0.62860	0.14050	0.1520*	
H9C	0.07470	0.50810	0.01780	0.1520*	
H10A	0.18810	0.65780	0.31830	0.1530*	
H10B	0.18120	0.48500	0.41480	0.1530*	
H10C	0.12270	0.62620	0.38370	0.1530*	
H11A	0.04780	0.36890	0.29760	0.0870*	
H11B	0.10950	0.24310	0.34380	0.0870*	

### Atomic displacement parameters (Å^2^)

**Table:**

	*U*^11^	*U*^22^	*U*^33^	*U*^12^	*U*^13^	*U*^23^
Cl1	0.1001 (11)	0.1304 (13)	0.0862 (9)	−0.0399 (9)	0.0299 (8)	−0.0167 (8)
S1	0.0632 (6)	0.0599 (6)	0.0404 (5)	−0.0007 (5)	0.0126 (4)	0.0005 (4)
O1	0.079 (2)	0.0732 (19)	0.0411 (14)	0.0032 (15)	0.0195 (13)	−0.0002 (12)
O2	0.083 (2)	0.0627 (18)	0.0608 (17)	−0.0105 (15)	0.0144 (15)	−0.0004 (14)
O3	0.0708 (19)	0.090 (2)	0.0481 (15)	0.0074 (16)	0.0166 (13)	0.0051 (14)
N1	0.063 (2)	0.068 (2)	0.0475 (18)	−0.0015 (17)	0.0115 (16)	0.0033 (16)
N2	0.063 (2)	0.084 (3)	0.0473 (18)	0.0104 (19)	0.0163 (16)	0.0035 (18)
C1	0.058 (2)	0.066 (3)	0.0420 (19)	−0.0019 (19)	0.0104 (16)	0.0019 (17)
C2	0.061 (2)	0.070 (3)	0.053 (2)	−0.001 (2)	0.0190 (19)	0.008 (2)
C3	0.063 (3)	0.065 (3)	0.064 (3)	0.003 (2)	0.021 (2)	0.008 (2)
C4	0.056 (2)	0.075 (3)	0.044 (2)	0.001 (2)	0.0119 (17)	0.0023 (19)
C5	0.067 (3)	0.086 (3)	0.059 (3)	0.003 (2)	0.025 (2)	0.015 (2)
C6	0.067 (3)	0.079 (3)	0.059 (2)	0.008 (2)	0.018 (2)	0.017 (2)
C7	0.058 (2)	0.069 (3)	0.044 (2)	−0.003 (2)	0.0143 (17)	0.0006 (18)
C8	0.074 (3)	0.070 (3)	0.053 (2)	0.008 (2)	0.023 (2)	0.002 (2)
C9	0.113 (5)	0.104 (4)	0.098 (4)	0.042 (4)	0.048 (3)	0.032 (3)
C10	0.125 (5)	0.090 (4)	0.097 (4)	−0.007 (3)	0.035 (4)	−0.030 (3)
C11	0.073 (3)	0.095 (4)	0.054 (2)	0.008 (3)	0.023 (2)	0.002 (2)

### Geometric parameters (Å, º)

**Table:**

Cl1—C11	1.778 (5)	C7—C8	1.536 (6)
S1—O1	1.439 (3)	C8—C9	1.516 (7)
S1—O2	1.427 (3)	C8—C10	1.540 (7)
S1—N1	1.602 (4)	C8—C11	1.522 (6)
S1—C1	1.762 (4)	C2—H2	0.9300
O3—C7	1.224 (5)	C3—H3	0.9300
N2—C4	1.424 (6)	C5—H5	0.9300
N2—C7	1.349 (5)	C6—H6	0.9300
N1—H1N	0.88 (3)	C9—H9A	0.9600
N1—H2N	0.88 (2)	C9—H9B	0.9600
N2—H3N	0.91 (6)	C9—H9C	0.9600
C1—C2	1.384 (6)	C10—H10A	0.9600
C1—C6	1.384 (6)	C10—H10B	0.9600
C2—C3	1.380 (6)	C10—H10C	0.9600
C3—C4	1.377 (6)	C11—H11A	0.9700
C4—C5	1.377 (6)	C11—H11B	0.9700
C5—C6	1.380 (6)		
			
O1—S1—O2	119.30 (17)	C9—C8—C10	110.6 (4)
O1—S1—N1	105.79 (17)	C9—C8—C11	110.6 (4)
O1—S1—C1	107.08 (18)	Cl1—C11—C8	113.6 (3)
O2—S1—N1	107.79 (18)	C1—C2—H2	120.00
O2—S1—C1	107.90 (19)	C3—C2—H2	120.00
N1—S1—C1	108.62 (19)	C2—C3—H3	120.00
C4—N2—C7	124.4 (4)	C4—C3—H3	120.00
H1N—N1—H2N	115 (5)	C4—C5—H5	120.00
S1—N1—H1N	110 (4)	C6—C5—H5	120.00
S1—N1—H2N	113 (4)	C1—C6—H6	120.00
C4—N2—H3N	113 (4)	C5—C6—H6	120.00
C7—N2—H3N	120 (4)	C8—C9—H9A	110.00
S1—C1—C2	120.7 (3)	C8—C9—H9B	109.00
C2—C1—C6	119.9 (4)	C8—C9—H9C	109.00
S1—C1—C6	119.4 (3)	H9A—C9—H9B	109.00
C1—C2—C3	120.4 (4)	H9A—C9—H9C	109.00
C2—C3—C4	119.6 (4)	H9B—C9—H9C	109.00
N2—C4—C3	122.1 (4)	C8—C10—H10A	109.00
N2—C4—C5	117.7 (4)	C8—C10—H10B	109.00
C3—C4—C5	120.1 (4)	C8—C10—H10C	109.00
C4—C5—C6	120.6 (4)	H10A—C10—H10B	110.00
C1—C6—C5	119.4 (4)	H10A—C10—H10C	109.00
O3—C7—C8	121.8 (4)	H10B—C10—H10C	110.00
N2—C7—C8	115.2 (3)	Cl1—C11—H11A	109.00
O3—C7—N2	123.0 (4)	Cl1—C11—H11B	109.00
C10—C8—C11	106.2 (4)	C8—C11—H11A	109.00
C7—C8—C11	110.4 (4)	C8—C11—H11B	109.00
C7—C8—C9	109.5 (4)	H11A—C11—H11B	108.00
C7—C8—C10	109.6 (4)		
			
O1—S1—C1—C2	−29.3 (4)	C2—C3—C4—N2	−179.9 (4)
O1—S1—C1—C6	154.6 (3)	C2—C3—C4—C5	2.7 (6)
O2—S1—C1—C2	−158.9 (3)	N2—C4—C5—C6	179.7 (4)
O2—S1—C1—C6	25.0 (4)	C3—C4—C5—C6	−2.8 (6)
N1—S1—C1—C2	84.5 (4)	C4—C5—C6—C1	0.8 (6)
N1—S1—C1—C6	−91.6 (4)	O3—C7—C8—C9	−1.1 (6)
C7—N2—C4—C3	42.6 (6)	O3—C7—C8—C10	−122.6 (4)
C7—N2—C4—C5	−139.9 (4)	O3—C7—C8—C11	120.8 (4)
C4—N2—C7—O3	−6.2 (7)	N2—C7—C8—C9	178.6 (4)
C4—N2—C7—C8	174.1 (4)	N2—C7—C8—C10	57.2 (5)
S1—C1—C2—C3	−177.4 (3)	N2—C7—C8—C11	−59.4 (5)
C6—C1—C2—C3	−1.4 (6)	C7—C8—C11—Cl1	−54.2 (4)
S1—C1—C6—C5	177.4 (3)	C9—C8—C11—Cl1	67.1 (4)
C2—C1—C6—C5	1.3 (6)	C10—C8—C11—Cl1	−172.9 (3)
C1—C2—C3—C4	−0.6 (6)		

### Hydrogen-bond geometry (Å, º)

**Table:**

*D*—H···*A*	*D*—H	H···*A*	*D*···*A*	*D*—H···*A*
N1—H1*N*···O2^i^	0.88 (3)	2.57 (5)	3.035 (4)	114 (4)
N1—H1*N*···O1^ii^	0.88 (3)	2.21 (4)	3.043 (5)	160 (5)
N1—H2*N*···O1^iii^	0.88 (2)	2.10 (4)	2.921 (4)	155 (5)
N2—H3*N*···O3^iv^	0.91 (6)	2.16 (6)	3.063 (5)	173 (5)
C3—H3···O3	0.93	2.49	2.896 (5)	106
C6—H6···O2	0.93	2.59	2.936 (5)	103
C11—H11*B*···O3^iv^	0.97	2.44	3.391 (5)	166

Symmetry codes: (i) −*x*+1, *y*+1/2, −*z*+1/2; (ii) −*x*+1, −*y*, −*z*; (iii) *x*, −*y*−1/2, *z*+1/2; (iv) *x*, −*y*+1/2, *z*+1/2.

## References

[bb1] Akkurt, M., Yalçın, Ş. P., Türkmen, H. & Büyükgüngör, O. (2010). *Acta Cryst.* E**66**, o1559–o1560. 10.1107/S1600536810020465 PMC3006765 21587803

[bb2] Altomare, A., Burla, M. C., Camalli, M., Cascarano, G. L., Giacovazzo, C., Guagliardi, A., Moliterni, A. G. G., Polidori, G. & Spagna, R. (1999). *J. Appl. Cryst.* **32**, 115–119.

[bb3] Asiri, A. M., Faidallah, H. M., Alamry, K. A., Ng, S. W. & Tiekink, E. R. T. (2012). *Acta Cryst.* E**68**, o2258–o2259. 10.1107/S1600536812028474 PMC3394043 22798908

[bb4] Farrugia, L. J. (2012). *J. Appl. Cryst.* **45**, 849–854.

[bb5] Idemudia, O. G., Sadimenko, A. P., Afolayan, A. J. & Hosten, E. C. (2012). *Acta Cryst.* E**68**, o1599. 10.1107/S1600536812018818 PMC3379209 22719407

[bb6] Parkin, S., Moezzi, B. & Hope, H. (1995). *J. Appl. Cryst.* **28**, 53–56.

[bb7] Poulsen, S., Bornaghi, L. F. & Healy, P. C. (2005). *Bioorg. Med. Chem. Lett.* **15**, 5429–5433. 10.1016/j.bmcl.2005.08.113 16213706

[bb8] Rigaku/MSC (2005). *CrystalClear* Rigaku/MSC, The Woodlands, Texas, USA.

[bb9] Sheldrick, G. M. (2008). *Acta Cryst.* A**64**, 112–122. 10.1107/S0108767307043930 18156677

[bb10] Spek, A. L. (2009). *Acta Cryst.* D**65**, 148–155. 10.1107/S090744490804362X PMC2631630 19171970

[bb11] Supuran, C. T. & Scozzafava, A. (2000). *Exp. Opin. Ther. Pat.* **10**, 575–600.

[bb12] Türkmen, H., Zengin, G. & Buyukkircali, B. (2011). Bioorg. Chem. **39** 114–119. 10.1016/j.bioorg.2011.02.004 21429552

